# Supplementary material for: Community cervical cancer screening: Barriers to successful home-based HPV self-sampling in Dabat district, North Gondar, Ethiopia. A qualitative study
Source: PLoS One. 2020 Dec 11;15(12):e0243036. doi: 10.1371/journal.pone.0243036 (PMC7732077; doi:10.1371/journal.pone.0243036)
Supplement: S1 File — (PDF) [file pone.0243036.s001.pdf]

**In-depth Interviews (IDIs) guide to understand barriers to home-based self-sampling.**

**Group participants:**

- Women who had previously participated in a community-wide home-based HPV self-sampling pilot study

Total participants of IDIs: 22

Items included in the in-depth interview guide

- Awareness about cervical cancer
- Beliefs about the causes and prevention of cervical cancer
- Awareness about cervical cancer screening
- Perception towards susceptibility and severity of cervical cancer screening
- Perception towards the benefits of participating in HPV self-sampling
- Belief in the early identification and treatment of cervical cancer
- Barriers to acceptance of HPV self-sampling
- Experiences of using the self-sampling instrument (Evalyn brush®)
- Husband/partner support and permission for the participants
- Barriers to the provision of a sufficient quality HPV testing
- Willingness to recommend HPV self-sampling to other women
- Suggestions of the participants to guarantee the success of home-based HPV self-sampling

**Focus group discussions (FGDs) guides to understand barriers to home-based self-sampling.**

**Group participants:**

- Women who had previously participated in a community-wide home-based HPV self-sampling pilot study
- Community health workers
- Women's development army leaders
- The sample collectors of the home-based HPV self-sampling pilot study.

Total Participants of FGDs: 25

Group Size for FGDs: 6-7

Number of FGD groups: 4

Items included in the FGD guide for women who had previously participated in a home-based HPV self-sampling pilot study, community health workers (CHWs), and women's development army leaders (HDALs)

- Awareness about cervical cancer and cervical cancer screening.
- Beliefs about the causes and prevention of cervical cancer
- Awareness on parts of women's body cervical cancer affects
- Indicators of a woman having cervical cancer
- Beliefs on early detection and treatment of cervical cancer
- Perception towards susceptibility and severity of cervical cancer screening
- Perception about where a woman should seek treatment for cervical cancer
- Willingness to participate in cervical cancer screening if given an opportunity
- How cervical cancer affects women's lives in the community

<u>Contents of FGD guide for the sample collectors of the home-based HPV self-sampling</u>

- Perception towards home-based HPV self-sampling
- Experiences towards how it was easy to explain the self-sampling procedure to the participants
- Perception towards the acceptance of self-sampling in the community.
- Experiences on how they overcame when it was difficult to women to understand the self-sampling procedures.
- Perception towards the challenges of women in providing a sufficient sample for HPV testing
- Challenges faced during visiting women's home for sample collection
- Perception towards husband/partner support to women's participation
- Suggestions on how to improve the quality of a home-based genital self-sampling for HPV self-sampling.
- Additional suggestions on how to improve the implementation of HPV self-sampling in the community.

## In-depth Interviews Guide Transcription

**Participants:**

- Women who had previously participated in community-wide home-based HPV self-sampling pilot study and provided quality samples for HPV testing.

- Women who had previously participated in community-wide home-based HPV self-sampling pilot study and did not provide sufficient samples for HPV testing.

**Interviewers: Ms.** S.W., T.N., and H.M.

**Duration of each interview:** 30-45 minutes.

**Interview setting and process**: Some of the women who had previously participated in home-based HPV self-sampling pilot study were traced based on the provision of quality samples for HPV testing and had scheduled for the in-depth interviews. The IDIs took place at the respondent's households. Before the discussion started, each woman was asked whether she can allocate time until the end of the interview. Women who had a job at hand were either waited until they finished the job or rescheduled for another convenient time. All IDIs were conducted in a confidential setting. After the overall information was given, the participants were asked their willingness to participate in the discussion as well as for tape recording. All of them agree on participation and tape recording after. The discussions, interviewers have used an interview guide, notebook, and tape record.

**Demographic characteristics of participants in the in-depth interview with women who provided a quality sample for HPV testing**

| S.No. | Participants code | Sex | Age range | Marital status | Educational Status | Parity |
|---|---|---|---|---|---|---|
| 1 | ESIDI01 | F | 35-40 | Married | No formal education | 1 |
| 2 | ESIDI02 | F | 30-35 | Married | No formal education | 5 |
| 3 | ESIDI03 | F | 50-55 | Married | No formal education | 6 |
| 4 | ESIDI04 | F | 40-45 | Married | No formal education | 6 |
| 5 | ESIDI05 | F | 35-40 | Married | No formal education | 2 |
| 6 | ESIDI06 | F | 45-50 | Married | No formal education | 8 |
| 7 | ESIDI07 | F | 30-35 | Married | No formal education | 7 |
| 8 | ESIDI08 | F | 35-40 | Married | No formal education | 7 |
| 9 | ESIDI09 | F | 25-30 | Married | Primary school | 2 |
| 10 | ESIDI10 | F | 25-30 | Married | Secondary school | 2 |

**Demographic characteristics of participants in the in-depth interview with women who did not provide sufficient sample for HPV testing**

| S.No. | Participants code | Sex | Age range | Marital status | Educational Status | Parity |
|---|---|---|---|---|---|---|
| 1 | ISIDI01 | F | 35-40 | Married | No formal education | 10 |
| 2 | ISIDI02 | F | 30-35 | Married | No formal education | 7 |
| 3 | ISIDI03 | F | 25-30 | Married | Primary school | 3 |
| 4 | ISIDI04 | F | 60-65 | Married | No formal education | 6 |
| 5 | ISIDI05 | F | 35-40 | Married | Primary school | 7 |
| 6 | ISIDI06 | F | 50-55 | Widowed | No formal education | 5 |
| 7 | ISIDI07 | F | 55-60 | Married | No formal education | 8 |
| 8 | ISIDI08 | F | 30-35 | Married | Primary school | 5 |
| 9 | ISIDI09 | F | 25-30 | Married | Primary school | 2 |
| 10 | ISIDI10 | F | 40-45 | Married | Primary school | 6 |
| 11 | ISIDI11 | F | 35-40 | Married | Primary school | 2 |
| 12 | ISIDI12 | F | 35-40 | Married | No formal education | 5 |

**Theme 1: Knowledge, perception, and beliefs toward cervical cancer and screening**

**Question 1: What can you tell me about cervical cancer?**

“This disease (cervical cancer) causes wounds to our uterus and finally damages our intestine.” (ISIDI05)

“It (cervical cancer) is a disease of the uterus that can be identified by causing discharge from woman's genitalia.” (ISIDI11)

“I don’t even know about this disease; I don’t have any word to say about it, this disease has come recently.” (ESIDI01)

“I don’t know about this disease that much, but I think it’s a disease caused by ‘Mitat’, sunlight rays, when woman urinates on the hot ground which expose her genitalia to evaporation coming out from that ground. I think it also affects women from not keeping personal hygiene.” (ISIDI10)

**Question 2: Could you tell me the local name for cervical cancer in this community?**

“We call it ‘Nekersa’ meaning cancer, but this disease has no other name in this community since it’s a new disease.” (ISIDI11)

“There is no local name given to cervical cancer here in our society, as others said it’s a new disease and we don’t have any more names for it.” (ESIDI06)

“We call it fistula disease; other people also name it “Neqersa”. (ISIDI05)

**Question 3: Which part of women’s body do you think cervical cancer can affect?**

“Huh, if she gets the disease, there will no body part that it leaves without affecting. A woman will feel pain all over her the whole-body parts once she gets this disease.” (ISIDI07)

“It affects the uterus of a woman. First, it affects a woman’s uterus and I think it will be changed to cancer later. It could also cause problems to the back and abdomen of a woman.” (ESIDI09)

“I think it affects a woman’s uterus. I have heard from people when they say a woman has uterine cancer, so I think it affects the uterus.” (ESIDI04)

“It affects a uterus of a woman, it involves her intestine and affects her mental health, and cause a woman to lose energy and she often doesn’t have the energy to work.” (ESIDI11)

**Question 4: What do you believe causes cervical cancer?**

“I don’t know what causes it, but I think it could be due to exposure to sunlight especially when a woman urinates on hot dry ground the vapor goes up to her uterus.” (ESIDI01)

“A woman gets this disease from smoke coming out from cow dung while cooking with open fire in the rural area, ‘Yegale Meret Lay Bemeshinat’ sunlight exposure while urinating on the hot ground and wearing dirty clothes.” (ISIDI03)

“In my opinion, this disease is caused by having sexual intercourse outside wedlock especially when husband divorce a woman she will go to another man and vice versa. This brings a lot of problems for a woman including this disease.” (ISIDI02)

“This disease is mainly caused by caused by exposure to sunlight rays 'Mich' and exposure of the genitalia to evaporation from urinating on the hot ground ‘Gerefta’ are the main cause of this

cancer among rural women. As you know we the rural women don't protect ourselves from sunlight when we travel, we urinate on the hot ground and sit on hot surfaces such as stones, there are what causes cervical cancer among rural women. So, if we think it can also be caused during delivery as we rural women give birth at home." (ESIDI 09)

**Question 5: In your opinion, what factors predispose women to cervical cancer?**

"I don't know, but in my opinion, we rural women usually use open fire for cooking everything, I think that is the one making us susceptible to this cancer." (ESIDI05)

"The first reason is lack of education on this disease. So, we are not aware of what brings cancer into our body. We are not much concerned about our body and we don't give ourselves enough care of what our body should get." (ISIDI07)

"Giving birth to many children, having sexual intercourse with many men and home delivery could predispose women to cervical cancer. Home delivery causes women's genitalia to be exposed to smoke coming out from using cow dung or woods during attending her delivery for long time as there is no electricity that we can use." (ESIDI09)

**Question 6: Could you describe the symptoms of cervical cancer?**

"Usually a woman having this cancer has a discharge which could even make her clothes to become wet from the back. Hence, when you see discharges over a woman's 'Qemis', skirt you can say the woman has cervical cancer." (ESIDI07)

"There will be irregular bleeding over a long time, urine incontinence, pain below a woman's waist, whitish discharge which could be seen over woman's cloth and causes shrinking to her clothes over her buttock." (ISIDI12)

"A woman having this cancer will have foul discharge, irregular bleeding, pain below the waist, abdominal pain, and it causes wounds over the uterus. When this disease affects the uterus, it will bleed and sometimes produce whitish discharge. It also causes severe pain during the menstrual bleeding." (ESIDI09)

**Question 7: Do you believe cervical cancer may be detected earlier and treated?**

“It could be identified if a woman goes to a health facility and see a doctor before it goes deep in her body and gets treated.” (ESIDI02)

“I think it could be treated if we visit a doctor when we see some of the indicators such as urine frequency, back, and abdominal pain. However, rural women often don’t go to a health facility unless we are severely sick. In addition, we use other available options such as seeking treatment from herbalists and we only go to doctors when the disease is worsened.” (ESIDI10)

“I don’t think the modern treatment can identify and treat this cancer, once a woman got the disease, it’s all about her, her fate will be death. I have never heard someone treated and cured of this disease. It’s really dangerous.” (ESIDI06)

“Yes, it’s treatable even in our places. For example, there are women in our locality who got treated and got better and now they are making their living. They are going to church though they have stopped having a baby.” (ISIDI09)

**Question 8: Could you tell me how to prevent cervical cancer?**

“We can prevent it through seeking treatment at a health facility and ‘Tsebel’ bathing in holy water.” (ESIDI01)

“As this disease is caused by urinating on hot ground “Gerefta” we need to protect ourselves from exposure our private parts to it, not urinating during sunny day in open field even when we go to market, we should avoid that. Also, while cooking it’s better if we avoid exposing our genitalia to smoke coming out from cow dung and other leaves, we can do that by wearing trousers. If we have an extra-marital sexual partner, I think using a condom can also protect us from having cervical cancer.” (ISIDI05)

“Limiting sexual intercourse to one partner. Keeping oneself neat, eating good food not just anything she got like unclean water and foods. But if you take good care of yourself you can prevent this cancer. Is it not because of the unclean water we are drinking and the food we are eating that causes cancer? So, taking good care will prevent all this thing before it reaches some late stage.” (ISIDI08)

**Question 9: In your community, where do you seek treatment for cervical cancer?**

"It could be treated by at hospitals; however, it could also be cured by 'Tsebel', bathing with holy water." (ESIDI05)

"She could only be treated at health facilities and if she goes to traditional medicine, I don't think she will get better. This disease is really bad and only good doctors can cure it." (ESIDI08)

"Going to "Tsebel' is another option for the treatment, it heals for those who have faith in God. However, not every holy water can heal it, there are known places for holy water where a woman could go and be free from the disease such as 'Werqishet', name of a religious place." (ISIDI11)

"Some women go and peptized with holy water as treatment. Also, I have seen women who went to magicians to get cured from this disease." (ESIDI08)

**Question 10: Do you think every woman should be screened for cervical cancer even if she is healthy?**

"If I am healthy, I don't want to go to a health facility, what I would say going there? So, unless I am sick, I wouldn't go for screening. I don't know why a woman should go for screening; is it when she gets sick?" (ESIDI03)

"In my opinion, a woman should be screened when she feels sick or see some indicators of the disease on her body. However, if she gets a chance to be screened at home as we did previously, she needs to be screened at any time because it's a great opportunity to know her health status at home." (ESIDI08)

"You see if you get this disease, you will lose 2 things, the first one is it affects your health since it will affect all your body, and the second one is your wealth as it's very expensive to seek treatment at referral hospitals which found in Addis Ababa. In addition, I don't want to be a burden to my family and community as this disease is very severe once you get sick. So, this is why I went for the screening before it affects my health." (ISIDI12)

"I think a woman should be screened to know as she is free from the disease. However, we rural women we don't go for screening; we keep waiting until the disease involves all of our body parts and severely sick. What can we do about it, we don't have knowledge about new diseases, also it's not easy to get to the hospital, there are also different expenses related with seeking care especially if the health professionals at health center tells you to other hospitals." (ESIDI06)

**Question 11: How do you perceive the severity of cervical cancer?**

“Since I have neither sick nor seen a woman sick of this cancer, I couldn’t describe how severe it’s.” (ESIDI04)

“It affects a woman’s life as it leaves a uterus without function, she could no more carry a baby in her womb. This is almost equal to death; can you imagine what life will be without having a baby. It also disturbs all of a woman’s life as it affects all her body especially the discharge is the one that makes her life miserable.” (ESIDI06)

“This disease is very dangerous. It causes a woman to suffer and die. It causes wounds to uterus and abdomen and finally kills her.” (ISIDI03)

“Woman can die from this disease unless she visits health facility so early, it’s a deadly disease. This disease is very bad as it causes foul discharge it affects woman both physically and psychology. Women having this disease always to hide from meeting other people as they are afraid, they can see the discharge.” (ISIDI10)

**Question 12: How could cervical cancer affect women’s lives in your community?**

“First of all, it damages a woman’s uterus as a result it could not carry anything for reproduction; the second one is it causes such a foul discharge and sometimes a woman experiences irregular bleeding. These all factors contribute to the reason why she wants to be alone then she will be susceptible even for a mental problem.” (ESIDI06)

“A woman having this cancer could be depressed as she may exclude herself from all social gatherings including from drinking coffee with her neighbors, relatives, and social insurance scheme “Idir” since she could feel embarrassed due to foul discharge caused by this disease. She could also think, her clothes are stained and wet, and it’s really shameful when you experience such a thing and people can see what has happened to you. Sometimes even if she doesn’t have any discharge, she may feel as she is having it and she avoids participating in any social gatherings.” (ISIDI08)

“A woman's neighbors will ask her by bringing foods and drinks and taking care of her children and help her to involve her in social life. However, she may feel not comfortable because of the nature of the disease. This disease is very dangerous as it causes bad smell. Due to this smell even,

your family could abandon you; you can’t expect help from your neighbors or society. It’s really difficult.” (ESIDI09)

“She could not feel comfortable to participate in any social event as she has that foul discharge. Even she could not happy to sleep with her husband due to the problems this disease could cause. You can imagine when flies go around you as a newborn baby due to that foul discharge, how she could join people’s gathering, even she could feel shy in her family. She may be bathing frequently but as this disease causes profuse discharge, I don’t think she could control it. It really damages her mental health.” (ESIDI10).

**Theme 2: Barriers to the success of HPV testing sample**

**Question 1: Could you explain your feeling as being a participant of home-based genital self-sampling?**

“In the first few days, people were talking bad things about the screening. They were saying the instrument has men sperm which could impregnate women, and some were saying it could cause disease by itself. However, after I had understood about cervical cancer and screening is one way of preventing this disease, I am very happy. Also, I have received my result and I am happy to be free from this disease.” (ESIDI09)

“As a participant of the home-based self-sampling, I am neither happy nor sad. I just participated because they told me as every woman in the community has participated in the screening.” (ESIDI05)

“I am not feeling happy for participating in home-based genital self-sampling because I have been waiting to receive my result, but they didn’t give me. Those who knew their result could be happy, but I am not. They told me as they would bring back my result, so where is it? I am still waiting but nothing has come yet. Maybe they have forgotten by this time” (ISIDI06)

“I really felt bad about it. First, I thought it was easy, but the experience was not easy. That time when the girls came, we were drinking coffee with my neighbors and I was embarrassed to go out and take the sample from my body. To be honest, I did it for the sake of respecting the girls who come to my home walking all long distance. I was not totally happy about it.” (ISIDI07)

**Question 2: Could you mention the importance of participating in home-based genital self-sampling?**

“It’s important for those who had the disease, they could follow treatment at an early stage and could cure this disease.” (ESIDI01)

“As the government brought this instrument to protect us from cervical cancer, I am happy to use it. Previously, we didn’t get any of screening in our community, now the government has brought it to our home, and it’s very important. People are paying even ETB 10,000 for treatment, but they are not getting better. This is a great opportunity for us to get this screening at our home.” (ISIDI05)

“It’s really important because it identifies whether you have this disease or not. It means a lot; like to identify as you will be living long or not.” (ESIDI08)

“We are doing it for your sake (sample collectors), I mean to help the collectors. You have an order to do so by the government so it’s your responsibility and we are doing it to pay respect for you not thinking we get advantage from it.” (ISIDI07)

**Question 3: What do you feel using the instrument (Evalyn brush®) for home-based genital self-sampling?**

“I don’t feel anything about it, I have used it as the girls described it for me to use.” (ESIDI01)

“When I applied the instrument to my genitalia, I felt an embarrassment, how come I insert this foreign thing to my genitalia, and it isn’t good at all. I don’t repeat this thing again” (ISIDI07)

**Question 4: Is there any cultural or religious issue related to using the instrument (Evalyn brush®) for home-based genital-self sampling?**

“The use of this self-sampling instrument is neither related to my religion nor to my culture as it’s about our health.” (ESIDI01)

“I don’t think there is any pressure from my culture or religion. In our religion, there are many things which are not allowed to participate in, but this screening is totally acceptable as it’s about our health.” (ESIDI10)

“We didn’t know before so there is no opinion about it. Our encounter with such a thing was for the very first time. So, the community and the religious leaders were not aware of it […] later all

the residents of our local community insulted me for participating in self-sampling and everybody was blaming me because I said yes to those girls collecting the sample, I really felt embarrassment for giving that sample." (ISIDI07)

"In the first place many women were not willing to participate in this screening program and some other were prevented by their husband as people were saying using the self-sampling instrument is like with sleeping with another man'. However, except the social misconception, there is no problem using the instrument from my religious side. But people still need education to understand this self-sampling instrument." (ISIDI12)

**Question 5: Did you face any challenge to use the instrument (Evalyn brush®) for self-sampling?**

"I didn't face challenge to collect the sample, but you know we have used this instrument for the first time, and I was feeling stresses as I didn't know this instrument before. It isn't easy to apply to your genitalia something that you have never seen in your life and never heard anything about it before. To be sincere with you, I was afraid to use the instrument considering many things people were talking about it especially about the instrument's containing men's fluid in it." (ESIDI06)

"There was no challenge I experienced to apply the instrument. The problem was more of personal bad feeling to use a foreign thing to my genitalia. I have never heard and seen such instrument even in the big hospitals and it was very difficult to me to convince myself and put a foreign material into my own genitalia, I felt totally strange thing in my life, hmmm." (ISIDI01)

"I didn't face any challenge to use the instrument because they showed me how to use it and I did it immediately." (ESIDI01)

"When I saw it at first, I thought it hurts my genitalia, but it didn't hurt. I am ok with using it as it's for our own health." (ESIDI10)

**Question 6: Did you have family support to participate in the self-sampling study?**

"My husband asked me about the screening, and I told him everything about the test. He was happy for my participation in the program. He appreciated the government for concern about women's health and bringing this new instrument at home. Also, he has encouraged me to participate in the screening program whenever it's available." (ISIDI03)

“I didn’t get any family support as they didn’t know as I have participated in sampling. Those collecting the sample come suddenly to my home and explained to me about the disease and asked me to give them a sample to identify as I am free from this disease.” (ESIDI01)

“I didn’t tell any of my family members because they came suddenly and told me to give them the sample after educating me and I agreed. So, I didn’t get a chance to discuss with my family.” (ISIDI06)

“We had discussed with my husband and even he was in the home when they came to collect the sample. They asked him whether he allows me to participate or not and he allowed me to participate. He just left after taking education with me, but he didn’t see the instrument.” (ESIDI10)

“How come I tell him as I did sampling on myself? It’s embarrassing to tell my husband since I inserted the instrument to my body and provided the sample, what a shame. Even I didn’t share it with anyone.” (ISIDI06)

“I didn’t tell him. He told me not to participate in the self-sampling. Even he refused the girls who were collecting the sample to enter our home. Then they left the compound that day and came back another time when he was not around and asked me whether I am willing to participate. They explained about the consequences of this disease and decided to give the sample.” (ISIDI11)

**Question 7: Do you recommend the method for another woman for cervical cancer screening?**

“I advise women to use the test because the instrument I used to collect the sample from my genital and it didn’t cause any pain while using, so if they think it could hurt, I will tell them as it doesn’t hurt. Maybe if you go and give a woman the instrument to use it, she could refuse to use it, but I can tell her from my experience.” (ESIDI01)

“I am advising many women to participate in the screening program to protect themselves from this bad disease and to get early treatment in case they have the disease as the government is providing both the treatment and screening for free. I was telling my neighbors and relatives to use this chance to be screened at their home that our government has brought.” (ISIDI02)

“I don’t recommend it for the other women, why should I recommend them to use the test? It has been such a long time and they were not able to give me my result. If they gave me the result, I

could say it's good.  However, they didn't give me the result though I was waiting since they collected it. So, I can't give a recommendation to any woman. Those who knew their status could recommend it for them."  (ISIDI06)

**Theme 3: Suggestions of the participants to guarantee the success of home-based HPV self-sampling.**

**Health education:** "We don't have enough knowledge about our health. Thus, we need to be supported with health education. In addition, we will be happy if we could get this instrument at nearby health facilities to check ourselves free from this disease." (ISIDI10)

**Male involvement in the screening programme:** "Our husbands also need to learn and support us; they need to be educated on this issue. There should be a program for men to give them education on cervical cancer and how to support their wife to participate in the screening program and give support in case she is sick from this disease." (ESIDI08)

**Lack of receiving the test result on time and linking those need follow-up and treatment to hospital:** "It's a good opportunity to take part in this screening program and I would suggest continuing for the future. In addition, I think it would be great if we can find this self-sampling instrument at nearby health post to check our health." (ISIDI11)

"One thing I want to suggest is that you should bring the result for those who didn't receive yet, some of them are in worry. The other thing is it's better if you do the screening again for those who refused to participate. These days most of them are ready to participate as they have realized it isn't causing harm to any one of the participants." (ISIDI05)

## FGD-1 Transcription

**Participants:** Women who provided insufficient sample for HPV testing.

**Moderator:** Ms. H.M.

**Starting Time:** 10:05 AM

**Ending Time:** 11: 27 AM

**FGD setting and process**: Seven women, who had previously participated in home-based HPV self-sampling and did not provide quality sample for HPV testing came together in Chila Satellite Health Post. They were sat in a circle in order everyone can easily discuss and see each other. After the overall information was given, the FGD participants were asked their willingness to participate in the discussion as well as for tape recording. They all agree on participation and tape recording. During the discussion FGD guide was used, note taking, and tape recording were done.

**Demographic characteristics of participants in the discussion**

| S.No. | Participants Code | Sex | Age range | Marital status | Educational Status | Parity |
|---|---|---|---|---|---|---|
| 1 | ISF01 | F | 45-50 | Married | Primary school | 7 |
| 2 | ISF02 | F | 35-40 | Married | No formal education | 4 |
| 3 | ISF03 | F | 40-45 | Divorced | Primary school | 2 |
| 4 | ISF04 | F | 45-50 | Married | Primary school | 7 |
| 5 | ISF05 | F | 45-50 | Widowed | No formal education | 6 |
| 6 | ISF06 | F | 35-40 | Married | No formal education | 6 |
| 7 | ISF07 | F | 35-40 | Married | No formal education | 6 |

**Theme 1: Knowledge, perception, and beliefs toward cervical cancer and screening**

**Question 1: What can you tell me about cervical cancer?**

"First, this disease (cervical cancer) caused when a menstruation cycle comes before the right time. Secondly, it is related to blood coming out from woman's uterus; and thirdly it causes a wound." (ISF01)

"I think it's a wound on the cervix of a woman." (ISF07)

"I don't know what it means, but I guess it's caused by a bad spirit. I am not educated, and no one taught me about it. So, I guess it might be a disease caused by sitting in dry hot places or when a person urinates in unclean places, that's is what I think" (ISF02)

"As my other friends said, I think it's caused by a bad spirit. You will have serious back pain and there will be a discharge from your uterus that's when you know you have the disease. The cancer

is a very serious disease which affects the life of the women very seriously. There is also no cure for it.” (ISF03)

“I perceive, cervical cancer as a very serious and very deadly disease. The government has also understood how the disease is serious and has launched a program to get us tested for cancer.” (ISF04)

“Let alone cancer on the uterus, even cancers on the bone are so deadly. Imagine what will happen when it’s on the uterus, that must be very deadly. Not getting the disease, even when I hear about it, I get frightened.” (ISF05)

**Question 2: Names given for cervical cancer in this community**

“Ok we call it ‘Nekersa’, cancer, the disease got many names, let God forbid it though.” (ISF05)

**Probe: Could you tell me other names?**

“It’s also called cancer. It’s not an easy disease for us and the government it’s trying to control the disease. But we pray it doesn’t come to us.” (ISF05)

“It’s also called ‘Nekersa’. Sometimes we also name it ‘Kintarot’, hemorrhoids.” (ISF06)

“It’s like a ‘Kintarot’, which affect your whole body even before you realize you had the disease. It’s when the disease gets into an advanced stage that a woman can know, and I think there is also a cure for it. After the people came here to get us tested, they told us a lot of story about this disease, we decided to take the test.” (ISF04)

“We also call it ‘Nekersa’, ‘kintarot’.” (ISF01)

**Question 3: Which part of the women’s body do you think cervical cancer can affects?**

“Primarily, it affects woman’s uterus. In addition, the disease affects heart, kidney, and her 12-body parts.” (ISF06)

“It affects all her 12 body parts. It even kills her. She doesn’t even live any longer since it kills so fast. There is even no way the woman knows she got cancer, so instead of going to health the facility, she tries traditional things at home and the disease will even progress more and she will die at home. Once you had it in your body it’s not going to prefer your body part it will kill you. It’s deadly.” (ISF05)

“Her whole body, heart, and intestine.” (ISF04)

“In most cases, if the cancer emerges from her intestine that mean it will spread into all part of her body mainly to the uterus. And the first place her uterus will be affected. She will have a lot of discharge and with a possibility of progressing to death.” (ISF03)

“I believe this cancer is a very dangerous disease and not after you get the disease even when we hear about the disease it sounds as a very serious disease.” (ISF02)

“She will stop eating and drinking food and will die from that. After the disease spreads in her body she couldn’t feed herself and she will die.” (ISF07)

“The first body part to be affected is the uterus. The second will be heart.” (ISF05)

**Question 4: Could you please tell us about the causes of cervical cancer?**

“It’s when you exposed to cold or hot weather. I think these are what causes the disease.” (ISF04)

“When you get exposed to extreme sunlight, when you sit on a hot stone and when you urinate on dry land and the vapors come up to your genitalia. When you wear dirty clothes, you will be exposed to this cancer.” (ISF07)

“I think people get this disease when there is sunlight and due to the sunlight, you sweat, and when you wear clothes which have been exposed to sunlight for a long duration. When there is sunlight and we sit on something hot and our body too gets hotter than the body will be exposed to this serious disease. I also think wearing a dirty cloth could bring this disease.” (ISF01)

“When sitting on a hot surface, when urinating on a dry place that’s when we get the disease. In addition, when we wear clothes spread in the sun for a long time.” (ISF03)

“I think the disease is caused by a bad spirit and urinating on a hot stone.” (ISF06)

“You get this disease when you wear clothes as soon as you collect them from outside after you spread them in sun for long duration. Stress and difficulties of life burden are also the other factors causing this cancer.” (ISF05)

**Question 5: In your opinion, what factors predispose women to cervical cancer?**

“The first thing which predisposes a woman for this cancer is urinating in a dry place, exposure to sunlight, wearing clothes left for long duration in sunlight, not seeking medical care as soon as she first saw the disease indicators are the main causes I can mention.” (ISF05)

“It was a great idea if a woman went to a health facility as soon as the disease started. The disease could have contained on its early stage. But when it gets to this cancer stage it will affect the blood and becomes more severe. The disease will spread and become ‘Kintarort’, it’s after this that it becomes cancer. So, going to health facility earlier has an advantage. (ISF04)

“Urinating in a dry place, not going to a medical facility as earlier as possible though it’s difficult as you know the life of a rural woman there will be a lot of tasks to take care and the situation doesn’t allow you to seek care immediately. So, you will be seeking care after disease advanced you became bedridden and after the disease has gone far.” (ISF03)

“It’s when a woman isn’t looking after her hygiene and not washing her genitalia every morning then that’s what causes the woman to have the disease. Though it’s also customary to visit health facility after the disease have gone far.” (ISF01)

**Question 6: Could you describe indicators of a woman having cervical cancer?**

“Although it won’t be immediately get confirmed, the disease will show an injury, swelling on the mouth of uterus.” (ISF05)

“There will be a discharge. The discharge will have blood or pus in it, and it will have a foul smelling. She will complain of heartache and headache. She will also have dizziness. This is all I can say.” (ISF06)

“This disease is a systematic disease so the whole-body part will be in pain. It’s also very embarrassing to go to a health facility too. It’s also making someone uncomfortable to talk about even if you get the disease. Then the disease will advance in its stage. Since the disease is embarrassing, it is even difficult to seek medical treatment.” (ISF04)

“Because the problem is in our genital area, we get embarrassed to speak about it. There will be a discharge. The discharge smells bad and contains pus in it. That’s all.” (ISF06)

“I think it affect the woman in her body unknowingly.” (ISF02)

**Probe: what are the symptoms?**

"There will be bleeding or discharge that's when the woman starts to complain. She may then go to the health facility. She either get her treatment or her disease may be unknown and lead her to death." (ISF02)

"She will have severe bleeding, back pain and due to the excessive bleeding her cloth will get dirty and she may feel like she had a fetus on her body and on her groin area there will be swelling. Then that's what she manifests." (ISF01)

**Question 7: Do you believe cervical cancer may be detected earlier and treated?**

"Yes, if the disease identified earlier before it gets into her inner body, she may have the chance to get treated. Even if she won't recover all in all, at least she can leave 10 to 15 years for her children. The difficulty is when we got the health facility after the disease advanced while trying some traditional stuffs [...] But I have to admit I never heard someone who got cancer and cured not sure if it's because it's recognized on the advanced stage." (ISF05)

"So, the woman will have a discharge which is different from the usual discharge because there will be blood and pus in it. So, if she gets tested earlier and knew her status, she may need to undergo some surgery and will be ok after then." (ISF06)

"I think if she goes to the health facility before the disease get disseminated to other parts of the woman body then she will recover from her sickness. But thinking she might get better tomorrow the disease might get into its advanced stage and the woman may die after a while." (ISF04)

"If cancer has grown from the uterus to the other parts of a woman's body it will be deadly. Early going to the health facility may help the woman to live a little longer but it can't cure her totally. The disease is incurable." (ISF03)

"The disease got no treatment and I never heard a person recovered after having cancer. Going to the health facility may help in reducing the pain for a while but that won't help any longer." (ISF02)

"I don't have a new idea. But going to the facility early, getting tested early will improve the life of a person and I believe she will get cured if she goes to the health facility earlier. But if we stay

longer in the house without treatment it will progress to the level where nothing can manage it." (ISF07)

"Once the disease is known that as it is a cancer then early treatment is good. But here in our community, you will go later. That's why I think we always regret that we should have gone early. If it's before the disease gets established there is a probability that she will recover from the disease. But if she couldn't go on time or I mean if she goes after getting sick then there is nothing the health professionals are going to provide her; she won't get anything." (ISF01)

**Question 8: What should a woman have to do to prevent cervical cancer?**

"Praying to God to protect you from such an evil disease, not urinating on a dry place, not wearing clothes which stayed longer on sunlight immediately, not sitting on everything they got, keeping oneself hygiene, and the best one will be praying." (ISF05)

"Not urinating on sunlight, not getting exposed to sunlight longer, avoiding sitting near smokes." (ISF06)

"Not sitting on a hot surface such as stone, sitting on a clean place, washing clothes with cold water, not getting exposed to extreme sunlight during the day." (ISF04)

"Not sitting on a hot stone, not wearing clothes immediately after we spread them for long duration outside." (ISF03)

"When we give birth at home when labor stays longer on us that's what exposes us to cancer. It's all about giving birth at home. At the health facility, we get treatment for everything. They will even stop bleeding with medication if it happens. So, going to the health facility is always helpful." (ISF02)

"Avoiding wearing clothes spread in sunlight for long duration, not getting exposure to the sunlight, exposure to smoke in the kitchen, when an early marriage happens. It's also important to put underwear as when we wear just one cloth it will expose us to a bad spirit." (ISF01)

**Question 9: Where do you think a woman gets treatment for cervical cancer?**

"We have to go to a modern health facility." (ISF06)

“It’s better to go to a health facility. We have seen so many consequences of going to a traditional healer. It’s one of the things making us die. We used to believe the traditional healers more than anything but it worth nothing. As soon as the disease starts if we got to the nearby health facility even if we don’t get long term cure, we will be ok for a while. In fact, a person with cancer will die as we hear in our society, but at least the health facility makes them live a little longer. After going to hospital, then we can go to holy water ‘Abuye Mikael’, the name of religious place, and the others. We don’t see ourselves dying.” (ISF05)

“It’s good if a person follows the holy water and pray to God with full heart and that will make the person get well. But when I say this, I am not forgetting the fact that it’s also very important to seek medical care. So, it’s also important to strictly follow the treatment ordered by the health professional. However, it’s also important to remember that following religious place is also important to recover fully from a disease. All these things are to prolong life because when the time comes there is no one who is going to escape death.” (ISF04)

“I also think better solutions are presented in a health facility. You won’t get anything from a traditional healer.” (ISF03)

“They (health facilities) don’t cut cancer and cure for once and instead they try to prolong life at the health facilities. But still, it’s better at the health facilities.  I never heard a person recovered from cancer all I hear is they passed away.”  (ISF02)

“Once the person gets cancer nothing can cure it. The place doesn’t matter for cancer. Our friends who went to higher health facilities even didn’t survive after she got cancer treatment. But for other disease health facilities are good to get treatment.” (ISF07)

**Question 10: Do you think every woman should be screened for cervical cancer even if they are healthy?**

“If she is a wise woman, she should do it when she is healthy. But in rural areas, we don’t have the awareness and we don’t get the time to go for such kind of test before we get the disease. We go late if not there is nothing good than going to a health facility earlier and knowing your status. But we don’t still trust the health facilities can recover our health so that’s why we decide to stay at home for 3 and 4 months until the disease make it’s a way to weaken us.” (ISF05)

**Probe: So, you are saying even getting tested is important even if she is healthy? Why is that?**

“Yes, because knowing her status before it is advanced will help her to prevent the disease. So, knowing yourself is the best to prevent future disease.” (ISF05)

“The problem is we delay a lot. For example, even me when I get sick, I will look for reasons not to go to the health facility. At times I will tell myself there is no food in the house and I should prepare for my family, my children are at school and no one is looking after the cattle and so many other reasons which prevent me from going to the health facility. Forget when I am healthy, I don’t even go the first moments I feel sick it has to get established and make me not to work my routine things that is when I think getting test helps, not while I’m healthy.” (ISF06)

“Hhhh going while healthy is unrealistic; believe me, I don’t even go when I am sick.” (ISF04)

“It’s good if the women get tested while she is healthy it will help her know what she has and don’t have. It will also help her from what disease she should protect herself. But getting tested while healthy in such a rural area is very unfamiliar.” (ISF03)

“Getting tested while healthy is good. But we go after the disease advanced and involve all our body. That’s when we decide to visit health facilities. Sometimes even I wonder if this is some of the cause why our sickness makes it difficult for the health professionals too to treat. So, when we are healthy it was our responsibility to get tested and know what is in our blood and not. It would have been great for us if we did the test before.” (ISF01)

**Question 11: If you have given an opportunity to cervical cancer screening are you willing to participate?**

“When did we receive even the result for the first one. I have already done the test twice I am not willing to undergo for the test anymore.” (ISF06)

“If I get sick, I will look for such a test.” (ISF01)

**Question 12: Could you describe how cervical cancer affects women’s lives in your community?**

“First when the disease starts it will start by swelling then she will start feeling the pain there will be so many problems where the disease may spread into her whole body until only the bone remains so that will have so many consequences on her life.” (ISF05)

**Probe: Do you think society is supportive of a woman having cervical cancer?**

“We try to support her. We frequently visit her at her home. We will wash her clothes and if possible, we will help her collecting money for her to get treatment, and if she has appetite, we prepare food at our home and give to her. We try to support each other.” (ISF05)

“The disease will make her lose appetite, it will also affect her intestine and she will have discharge and it will lead her to death.” (ISF03)

“When I woman gets discriminated and excluded by her family and friends, she may lose her family, she may divorce.” (ISF06)

**Probe: what about a woman with the disease, will she be happy to participate in social life?**

“She is already affected, unless her mental condition gets deteriorated, she will hear us out and do what we advise her.” (ISF05)

“Because she may have foul-smelling discharge, she may feel embarrassed. She may think that we try to exclude her because of her situation and may try to not show up to us. But we try to comfort her in all these situations” (ISF03)

“She may get embarrassed with her situation and that smell and may try to avoid us anticipating that we may not become supportive.” (ISF01)

**Theme 2: Suggestions of the participants to guarantee the success of home-based HPV self-sampling.**

**Question**: is there anything you want to say finally?

“We will be happy if the health professionals provide us more information on this issue. We like if we learn more about this disease whenever they could make time.” (ISF05)

“We love if they send us the treatment or any medication that prevent the disease. it will be great if the government send us the drugs as they send to us the contraceptives.” (ISF03)

"It will be great if people who are educated come to us and teach us about the disease and we had the chance to know more about the disease. It's also great if we also teach others who are not aware. (ISF01)

## FGD-2 Transcription

**Participants:** Women who provided quality sample for HPV testing.

**Moderator:** Ms. H.M.

**Starting Time:** 11:20 AM

**Ending Time:** 12:28 PM

**FGD setting and process**: Six women, who were participated in home-based HPV self-sampling and provided a sufficient sample for HPV testing came together in Chila Satellite Health Post. They were sat in a circle in order everyone can easily discuss and see each other. After the overall information was given, the FGD participants were asked their willingness to participate in the discussion as well as for tape recording. They all agree on participation and tape recording. During discussion FGD guide was used, note taking, and tape recording were done.

**Demographic characteristics of participants in the discussion**

| S.No. | Participants Code | Sex | Age range | Marital status | Educational Status | Parity |
|---|---|---|---|---|---|---|
| 1 | ESF01 | F | 35-40 | Married | Primary school | 6 |
| 2 | ESF02 | F | 30-35 | Married | No formal education | 6 |
| 3 | ESF03 | F | 25-30 | Married | Secondary school | 2 |
| 4 | ESF04 | F | 25-30 | Married | No formal education | 6 |
| 5 | ESF05 | F | 25-30 | Married | No formal education | 4 |
| 6 | ESF06 | F | 25-30 | Married | Primary school | 4 |

**Theme 1: Knowledge, perception, and beliefs toward cervical cancer and screening**

**Question 1: What can you tell me about cervical cancer?**

"It's difficult, but I think it causes wound to the neck of the uterus and results in foul discharge which comes out from the uterus." (ESF01)

"This disease causes wound to the uterus and foul discharge from genitalia." (ESF02)

"Cervical cancer causes discharge from the woman's genitalia."  (ESF03)

"Cervical cancer affects all our body system and causes death." (ESF04)

"It's caused when a woman exposes her genitalia to sunlight rays." (ESF05)

"This disease causes pain and bleeding especially when you walk long distance." (ESF06)

**Question 2: Could you tell me the name of cervical cancer in this community?**

"I have heard this cancer for the first time when you came and gave us health education during the genital self-collection; what I know is the name you told as, cervical cancer. However, I know breast cancer, bone cancer, and brain cancer, but I don't know even this cervical cancer before you come to our community to give us health education and sample collection." (ESF05)

"I have heard for the first time when you come to our community for screening. Even the word cancer come recently, and we don't know it previously." (ESF02)

"Some people call it fistula disease."  (ESF01)

**Question 3: Which part of women's body do you think cervical cancer can affect?**

"I think it affects woman's uterus and causes discharge and she couldn't give birth." (ESF01)

"It affects her uterus and abdomen and causes infertility." (ESF02)

"It affects her uterus and she will be very tired." (ESF03)

"It affects her uterus and it causes pain to all her body particularly to a woman's intestine." (ESF04)

"It affects all her body part and finally causes death." (ESF05)

**Question 4: What do you believe as the causes of cervical cancer?**

"It's caused by sitting on hot surfaces such as stone, but it could also be caused by sitting on a cold stone." (ESF01)

“It could be caused when woman’s genitalia are exposed to dirty things and having sex with man with older age, disproportional size of sex organs of male and female while having sexual intercourse. For instance, if a girl under 18 years of age have sexual intercourse with an adult male, it causes cervical cancer.” (ESF02)

“Sitting on a hot stone, when woman’s genitalia are exposed to smoke coming out from cow dung when cooking, early marriage, and getting pregnant at a small age.” (ESF03)

“When woman’s vagina is exposed to sunlight, cooking for a long time with firewood with open fire.” (ESF04)

“When a woman lifts heavy loads, fall down suddenly, and get injured and wounded it could be the initial point for this cancer causes unless we seek treatment at a health facility before it gets complicated.” (ESF05)

**Question 5: In your opinion, what factors predispose women to cervical cancer?**

“In my opinion, open fire cooking with firewood or cow dung and heavy work are the initial causes of this cancer.” (ESF03)

“Sexual intercourse could predispose a woman as sometimes woman's genitalia could not carry a man's fluid for a long time as it causes a problem to her uterus, will be a chance to be changed to cancer. In another way, when a woman cooks with open fire the smoke comes out from the firewood or cow dung could make her susceptible her for this cancer as it exposes our genitalia to smoke.” (ESF05)

“Having many family members as it could cause a lot of stress and giving birth to a lot of children could put a woman at increased risk to get this cancer.” (ESF06)

**Question 6: Could you describe the symptoms of cervical cancer?**

“I don’t know because I have never seen a woman sick from this type of disease.” (ESF01).

“I suspect a woman as having cervical cancer when I see discharge which causes shrink to hear cloths over her buttock.” (ESF02)

“Unless she told us as having cervical cancer, it isn’t easy to identify a woman sick from cervical cancer. But usually, there will be whitish and bloody discharge over a woman's clothes having this

disease. In addition, when a lady has prolonged irregular bleeding or has a menstrual period for a long duration we should suspect as having this cancer." (ESF04)

"There will be a frequent foul discharge and bleeding coming out from a woman's genitalia which prevents her joining with other people." (ESF05)

**Question 7: Do you believe cervical cancer may be detected earlier and treated?**

"In my opinion it could be cured if a woman goes to a health facility, follow treatment, and stop having sexual intercourse until she cured from the disease." (ESF01)

"If she follows the treatment she could be cured of the disease." (ESF02)

"Most of the time we delay going health facility because we think it could be improved at home and it could also be due to lack of money. However, if a woman goes to a health facility at the early stage of this disease before it includes her intestines and follows treatment, she will be cured of it." (ESF03)

"If she goes to a health facility by the time it starts her, she could be cured. However, if she waits until the disease involves other parts of her body and until she sees discharges from her genitalia, I don't think she will be cured of this disease even if she visits big hospitals." (ESF05)

**Question 8: Could you tell me how to prevent cervical cancer?**

"A woman needs to protect herself from sunlight exposure particularly she should not urinate in hot dry land which causes evaporation. Also, screening as you told us when you came for genital self-sampling could prevent this disease." (ESF01)

A woman should protect herself from 'Mitat' sunlight exposure, should avoid wearing clothes spread in sunlight for a long duration, avoiding sitting over hot chairs, and avoiding forced sex." (ESF03)

"Avoiding early marriage, avoiding sitting over a hot chair, and avoiding exposure of genitalia to smoke while open fire cooking." (ESF04)

**Question 9: Where do you think a woman should get treatment for cervical cancer?**

"A woman can go to holy water even after going to the hospital if she didn't get cured of this disease." (ESF06)

"A woman having cervical cancer needs to go to a health facility and most of the time she will be referred to Addis Ababa, Black Lion Hospital. In addition, she could also go to holy water 'Tsebel' if she could not get any solution from the hospital. Also, a woman can go to traditional healers, but she may not get any improvement from local people." (ESF04)

**Question 10: Do you think every woman should be screened for cervical cancer even if they are healthy?**

"Yes, to identify herself as free from this disease, every woman should be screened as you have told us last time." (ESF05)

"Last time when you brought this screening at our home, many women who didn't know as they were having this cancer were identified as having this disease. Therefore, if possible, it's great if every woman gets screened to identify themselves from this disease." (ESF06)

"If I am not sick how I could go to health facility but if people come to my home as you did last time I may participate in screening, unless I don't want to go." (ESF01)

"Unless I can see some of the manifestations on myself, I won't go to a health facility for screening, how can I go?" (ESF03)

**Question 11: If you have given an opportunity to cervical cancer screening are you willing to participate?**

"Yes, I will use any opportunity to get tested to identify myself from this disease." (ESF05)

"If I have no indicators as I have the disease, why I need to be screened? I don't think I will go for it." (ESF01)

"If I don't feel sick, I won't go for screening, but when I am sick, I will run to a health facility for the screening. How I will go to a health facility while I am feeling well? I will never go." (ESF03)

**Question 12: Could you describe how cervical cancer affects women's lives in this community?**

"Since there will be a discharge coming out from a woman's genitalia, it affects her life and maybe she could no sleep with her husband, which cause embarrassment. Due to this reason she will be

excluded from the society or she excludes herself from society and her mental will be affected as she feels lonely." (ESF02)

"Due the discharge, she may not meet with her friends and relatives, which affects her mental." (ESF04)

**Question 13: Do you think society is supportive of a woman having cervical cancer?**

"We ask her how she feels but when she is severely sick of this disease, we should tell her to go for a treatment at a hospital." (ESF04)

"It depends, if she is severely sick, we can't take her with us when we go to social ceremonies. But she may not feel comfortable when she joins us because she worries about the foul discharge. Even if she might not have any discharge, she could afraid as this disease affects a woman's mental badly." (ESF06)

**Theme 2: Suggestions of the participants to guarantee the success of home-based HPV self-sampling.**

**Question: Is there anything you want to say finally?**

"Educated people are good and we are always ready to participate in the screening to keep ourselves healthy. We expect from you to come and screen us as you did last time. Also, you need to explain it in the language that we can understand. Sometimes we, a rural woman could say, I don't want to participate because of we have limited understanding about it, so you need to explain the advantages and the consequences of not participating in the screening." (ESF05)

"It's about our lives, previously you told us to be screened and we did it. If you are telling us to repeat the screening, we will do that. But as this disease is very bad, we need frequent health education on this disease. Also, those women who were not willing participants in screening previously should be educated to screen as this disease affects woman life." (ESF06)

## FGD-3 Transcription

**Participants:** Community Health Workers (CHWs) and women's development army leaders (WDALs).

**Moderator:** Ms. H.M.

**Starting Time:** 2:15 PM

**Ending Time:** 3:37 PM

**FGD setting and process**: Six women (one community health worker and five women's development army leaders) came together in Chila, Satellite Health Post. They were sat in a circle in order everyone can easily discuss and see each other. After the overall information was given, the FGD participants were asked their willingness to participate in the discussion as well as for tape recording. They all agree on participation and tape recording. During the discussion FGD guide was used, note taking, and tape recording were done.

**Demographic characteristics of participants in the discussion**

| S.No. | Participants Code | Sex | Age range | Marital status | Educational Status | Parity |
|---|---|---|---|---|---|---|
| 1 | HEDA01 | F | 40-45 | Married | Primary school | 0 |
| 2 | HEDA02 | F | 40-45 | Widowed | Primary school | 2 |
| 3 | HEDA03 | F | 25-30 | Married | Secondary school | 2 |
| 4 | HEDA04 | F | 50-55 | Married | Primary school | 8 |
| 5 | HEDA05 | F | 45-50 | Married | Primary school | 4 |
| 6 | HEDA06 | F | 35-40 | Married | Primary school | 4 |

**Theme 1: Knowledge, perception, and beliefs toward cervical cancer and screening**

**Question 1: What can you tell me about cervical cancer?**

"It's a disease that causes swelling and wound to the cervix of a woman which could be treated if a woman visits health facilities before it becomes severe." (HEDA01)

"This disease is common among the women, but it also affects men. It causes pain around genitalia and back of the body. It could only be identified at the health facility before that we could not know whether we had the disease or not." (HEDA04)

“In my understanding cervical cancer is the disease of the uterus that affects the part of woman's intestine over time.” (HEDA02)

“Cervical cancer is a disease that could be characterized by swelling, itching of the cervix, then it becomes wounded, these things could be seen on the cervix.” (HEDA03)

**Question 2: Names given for cervical cancer in this community**

“It’s called a fistula disease.” (HEDA01)

“In the rural community, the name given for cervical cancer is ‘Nekersa, Kintarot’.” (HEDA05)

“For the first time I have heard the name of this cancer when they did the screening last year, it’s a new disease which came recently. The common cancer names I am familiar with are breast cancer, bone cancer, and uterine cancer.” (HEDA3)

**Question 3: Which part of the women’s body do you think cervical cancer can affects?**

“It affects her genitalia then it goes to her abdomen. Since it causes wound to the opening of the uterus it causes infertility so she could not become pregnant.” (HEDA01)

“It affects women’s uterus and blood vessels.” (HEDA04)

“This disease affects the uterus and as the uterus is found in the center of women’s body it goes to the other parts of her body. When it expands to other parts of the body she could not work, eat well and she will die of it.” (HEDA02)

“First of all, our uterus is found at the center of the abdomen and as the women are working hard it can easily go to her breast, then to her head. However, our uterus is the one which affected most.” (HEDA03)

“Cervical cancer only affects the uterus, but it could cause pain to the other parts of the body.” (HEDA05)

**Question 4: What do you believe as the causes of cervical cancer?**

“This disease comes from smoke coming out when we urinate on dry and hot ground, ‘gerefta’, and exposure to sunlight rays ‘Mitat’, sun allergy.” (HEDA01).

“There is no known factor that causes cervical cancer, but from what people say, it comes from delivering many children, labor during delivery, urinating on hot places, lack of keeping personal hygiene particularly while home delivery. When woman labor at home it takes a long time to deliver, the genitalia of a woman could be touched with unclean hand and may be materials, this believed to cause cervical cancer.” (HEDA5)

“As my colleague has said earlier, this disease is caused by ‘Mitat and Gerefta’. when we expose our genitalia to a vapor come while urinating on dry hot land it goes up to a woman's uterus and causes cervical cancer.” (HEDA02)

“In the rural area, we are exposed to this disease as the result of using cow dung for open fire cooking ‘Yekubot Tis’. The smoke coming out from it directly goes to a woman's genitalia, and cause cancer to the uterus. In addition, we use a skirt (Kemis) while cooking and our genitalia is exposed to the smoke coming out of the dried cow dung and it enters our genitals, ‘Metaxen’ that is the one causing this cancer. The other thing I think causing this cancer is holding urine for a long time ‘Yeshinx max’; when a woman holds urine for the long period of time it will affect the uterus and there will be a chance for the uterus to develop cervical cancer.” (HEDA03)

“It’s caused by urinating on very hot dry ground, when we urinate on the hot dry land it evaporates to our genitalia, ‘Yegale Meret lay bemeshinat’ and exposing once genitalia to the sunlight rays ‘Gerefta’. The other thing is holding urine for long period, when we hold urine it will be accumulated in the uterus and causes cancer to the uterus.” (HEDA04)

“In my opinion, most of the time rural women don’t keep their personal hygiene particularly when they are on the menstrual cycle and when they urinate on the hot ground while menstruating it causes cancer to a woman.” (HEDA06)

**Question 5: In your opinion, what factors predispose women to cervical cancer?**

“When a woman will be in prolonged labor her genitalia will be at the risk of being touched repeatedly and infected particularly when a woman give birth at home. When a woman gives birth at home, there are many things they do including firing dry cow dung which smoke and could result in cervical infection.” (HEDA05)

“Lack of check-up, if she goes to a health facility on time this disease could be identified at an early stage, if she goes to a health facility after it becomes severe, it affects her life including causing death.” (HEDA03)

“When woman labor and give birth, it hurt her uterus and the wound will be changed to cancer. So that giving birth causes a woman to get this cancer.” (HEDA04)

“In my opinion, when a woman gives birth there will be a lot of discharge coming out of her body, this discharge including blood which spills out of a woman uterus will make her susceptible for cervical cancer.” (HEDA01)

“In addition to what I have said earlier, having multiple sexual partners predispose a woman for cervical cancer. For instance, sex workers sleep with many sexual partners and they are more susceptible to cervical cancer, having more than one sexual partner could predispose a woman for cervical cancer.” (HEDA05)

“Prolonged labor puts a woman a risk to develop this disease; if she isn’t taken to the health facility on time during labor, it can affect her genitalia which later could be changed to cancer.” (HEDA06)

**Question 6: Could you describe indicators of a woman having cervical cancer?**

“In a rural area we know a woman having cervical cancer by looking at her skirt from behind; a woman having cervical cancer has a discharge that can be seen on her skirt and usually if we see a woman’s dress which is wet and shrink around her lower part we say she has uterine problem. This kind of discharge is quite different from other discharges, it will be like white discharge on a woman’s dress and her skirt wrinkles especially when she stands from sitting.” (HEDA01)

“When we hear that a married woman prevents her husband to sleep with her, we suspect that a woman has cervical cancer.” (HEDA06)

“We can guess by looking at her clothes from behind, but we don’t know unless she tells us, how could we know? Even we can’t be sure from looking at discharge on her skirt, it could be another thing. We also guess as a woman having cervical cancer when she ties her waist. In my opinion, she ties her waist to get relief from the pain she feels from her uterus and her back while sitting and standing.” (HEDA02)

“A woman with this cancer has a smelling discharge coming out of her uterus, which prevents her from having sex with her husband. It causes an itching sensation around her genitalia and makes her body full of wounds particularly around her vagina.” (HEDA03)

“It is believed that a woman having pain below her umbilicus, foul discharge, severe back pain, internal burning sensation; when a woman complains all these, we suspect a woman having cervical cancer. In addition to these sign and symptoms, there will be frequent abnormal bleeding that is quite different from the menstrual cycle.” (HEDA05)

“It isn’t easy to identify women having cervical cancer, they never talk to another person as they have cervical cancer, and how they will talk about it? However, as it has been mentioned we can identify a woman having cervical cancer by looking at her clothes from the back and you can see whitish discharge or bloody discharge that causes her skirt over her buttock to fold. Due to these discharges, you can’t approach her as it causes very bad smelling. It also causes a woman so thin and leaves her without flesh; it causes all her body to dry like dried wood, this is due to she is losing a lot of fluid from her body either as a pussy discharge or as blood.” (HEDA04)

**Question 7: Do you believe cervical cancer may be detected earlier and treated?**

“If a woman goes to the health facility on time a doctor can see cancer and if she gets early treatment she could be saved. However, after it controls all her uterus, even if she goes to a doctor, she can’t get cure from this disease.” (HEDA01)

“Cervical cancer could be diagnosed and treated early if it’s detected at stages I and II, but at stage III, when it controls all her uterus it could not be cured. However, in my opinion, in the first stage as it only affects the cervix it could be treated with medicine or operation.” (HEDA05)

“If a woman treated at an early stage, she could be cured of the disease unless going to the health facility after it controls the uterus a doctor can’t give her solution.” (HEDA03)

“In my opinion, if a woman goes to see a doctor immediately after she realizing something is wrong with her uterus she can be treated and healed from this disease.” (HEDA06)

“I think if a woman goes to a health facility within 2 weeks after she realized having itching sensation and wound over her cervix, she could be treated at a health facility even if she may not be cured of this disease.  If she follows the treatment well, she could live longer.” (HEDA02)

**Question 8: Could you tell me how to prevent cervical cancer?**

"To prevent cervical cancer, a woman should avoid urinating where she could be exposed to sunlight, keeping personal hygiene, not exposing her genitalia to dust, since a rural woman doesn't wear trousers they need to keep away their genitalia from dust particles and keep it clean. I think using a toilet is also one of the prevention methods as a woman couldn't be exposed to sunlight when she uses it." (HEDA01)

"In my opinion a woman need to protect herself from urinating on hot ground and when she is in the menstrual cycle it's better to use a toilet to avoid exposure to sunlight and not to sit on hot surfaces such as stone; keeping her neatness by changing sanitary pad daily while a woman is on her menstrual cycle as it's the most critical time to cause cervical cancer when we are exposed to sunlight." (HEDA02)

"As they have said, to prevent this cancer we should urinate under a shadow to avoid sunlight exposure and the second one we need to keep our neatness. We also need to taker from exposing our genitalia to smoke while cooking and changing the clothes that we wear while cooking or it's important to wear trousers to avoid exposure of our genitalia to the smoke from cow dung and bahirzaf leaf." (HEDA03)

"The main way of prevention is through keeping cleanliness and delivering at the health institution. The other important prevention method is by having a single sexual partner in life. If a woman has a husband, she should be faithful to him and for those who didn't have a husband they also need to have a single sexual partner. The other thing is if a woman is a sex-worker it's better if she uses condom consistently." (HEDA05).

**Question 9: Where do you think a woman gets treatment for cervical cancer?**

"If a woman has this disease, she should go to health facility for treatment. I don't think a traditional medicine cure her of the disease." (HEDA06)

"In my opinion, a woman should go to big hospitals such as Gondar referral Hospital and Black Lion hospital in Addis Ababa." (HEDA02)

"First, a woman should go to a nearby health facility to identify whether she has the disease, then she needs to go to referral hospitals to get treatment." (HEDA03)

“Cervical cancer can only be treated at hospitals; traditional medicine couldn’t cure this disease as they use different leaves to treat a disease. But holy water can cure this disease if we have faith in it.” (HEDA05)

“I believe cancer could be treated only at a health facility.” (HEDA01)

**Question 10: Do you think every woman should be screened for cervical cancer even if they are healthy?**

“A woman should go to a health facility to identify whether she is healthy or not. However, most of the women don’t go for screening because they fear to show their genitalia to a doctor. Even many women feared to provide the genital sample in their bedrooms, and they returned the instrument for self-sampling without using it. They went to their bedrooms and pretended as they have used the instrument, but some women didn’t use it.” (HEDA01)

“If I am healthy and don’t have any indications of cervical cancer, why do I go for screening? If I am healthy, I don’t want to go for screening.” (HEDA02)

“I think it’s good to be screened, but it would be better to go for screening if a woman suspects herself having the disease. If I don’t have any indicators, what do I say to the doctor when he/she ask me why I am there? So, in my opinion, to go for screening, a woman should see at least some symptoms of abnormality. We go for screening when we fell sick.” (HEDA03)

“I think it isn’t possible to identify whether a woman has cervical cancer unless she goes for screening, so every woman should go for it to identify herself. I can’t be sure as I’m not having this disease unless screened by giving my sample.” (HEDA05)

“I want to go for screening as you can’t identify yourself unless you are screened. Last year many women have told us they have cervical cancer but before that, they don’t know as they do have this cancer. So, screening is the only method to identify yourself as you are free from the disease. I want to go for screening before I get sick, to identify my status.” (HEDA06)

**Question 11: If you have given an opportunity to cervical cancer screening are you willing to participate?**

“If I am given a chance for screening, I will go for it. The government is giving us a chance to be screened being at our home. So, I am willing to participate in a screening.” (HEDA01)

“Previously, I have taken part in screening and if it comes again, I will participate as I want to know my status.” (HEDA02)

“Now it’s a blessing to use a screening instrument at our home, and I want to use it to know my status. If they tell me I am healthy it’s a blessing but if they tell me as I am having the disease I will go for further treatment.” (HEDA03)

“If we get the chance of screening, we need to accept it because we didn’t go for screening previously. If I don’t have the disease it’s good and if I have, I will continue for treatment.” (HEDA05)

“Since this disease is very serious, I want to identify myself as I am not having the disease.” (HEDA04)

**Question 12: Could you describe how cervical cancer affects women’s lives in this community?**

“This disease affects a woman’s life in different ways. In the first place since this disease is very serious and painful, she suffers and can’t do any work. The second one is because of this disease causes very foul discharge she ends up in divorce and may not get anyone who will be taking care of her including her neighbors and relatives. Due to the disease, she will be excluded from social life, this causes a psychological problem to her.” (HEDA05)

“This disease will affect her body and she will end up in death. Her children will be orphan, and they won’t get anyone who helps her. So, this disease affects both the woman and her family. The society may not exclude her, but she may not feel comfortable to participate in social life, as she could fear the smelling could disturb others.” (HEDA02)

“It causes a severe psychological impact as her husband divorces her and exclusion from social life. However, as I didn’t have of a woman having this disease in my neighbor I couldn’t able to explain how people could treat her. But in my opinion, I won’t exclude her from any social activity but could exclude herself as she may not feel comfortable due to the foul of the discharge.” (HEDA03).

“If she didn’t go health facility on time, it will affect her abdomen and she couldn’t eat well and finally, it kills her. We don’t exclude her from the community, but we advise her to go to a health facility.” (HEDA01)

“From my experience, there was a lady who was sick of cervical cancer. She had very offensive discharge coming out from her genitalia, because of this discharge not only social and relative exclusion but her husband and child abandon her.” (HEDA05)

“Since cervical cancer isn’t communicable from one person to another, we need to help her, we can drink coffee together, and we may not feel comfortable in helping her when she became bedridden. In addition, she could exclude herself from social life as she couldn’t feel comfortable due to the problems the disease caused to her. As long as she lives, we need to support her as she needs more psychological support from her neighbors and friends.” (HEDA06)

**Theme 2: Suggestions of the participants to guarantee the success of home-based HPV self-sampling.**

**Question: Is there anything you want to say finally?**

“It would be very important if we could get the self-sampling instrument at a nearby health facility. Any woman interested to check herself should access to the screening instrument and as once started we believe the government will make continue providing this service. There are also many women who didn’t get this chance and I they also need it. In case it’s difficult for the government to provide this instrument for woman at home, even if we can access it at nearby health facility, we can use it.” (HEDA05)

## FGD-4 Transcription

**Participants:** Sample collectors of home-based HPV self-sampling.

**Moderator:** Mr. B.S.

**Starting Time:** 1:30 PM

**Ending Time:** 3:03 PM

**FGD setting and process**: Five women who were participated in sample collection of home-based HPV self-sampling and one man who was supervising the sample collectors came together in the health center. They were sat in a circle in order everyone can easily discuss and see each other. After the overall information was given, the FGD participants were asked their willingness to participate in the discussion as well as for tape recording. They all agree on participation and tape recording. During the discussion FGD guide was used, note taking, and tape recording were done.

**Demographic characteristics of participants in the discussion**

| S.No. | Participants Code | Sex | Age range | Marital status | Level of education | Parity |
|---|---|---|---|---|---|---|
| 1 | DC01 | F | 20-25 | Single | Tertiary school | 0 |
| 2 | DC02 | M | 45-50 | Married | Tertiary school | 4 |
| 3 | DC03 | F | 20-25 | Single | Tertiary school | 0 |
| 4 | DC04 | F | 25-30 | Single | Tertiary school | 0 |
| 5 | DC05 | F | 25-30 | Single | Tertiary school | 0 |
| 6 | DC06 | F | 25-30 | Married | Tertiary school | 1 |

**Theme 1: Barriers to acceptance of HPV self-sampling**

**Quetion 1: How do you feel about the study of home-based genital-self sampling for cervical cancer screening?**

“It was really important for women who participated in the screening because many women who screened could have missed this opportunity as most of the women were not come to health facility for screening unless they are severely sick. This screening program gave them a chance to be screened at their home particularly for those who couldn‘t come to a health facility.” (DC01)

“This study was implemented in ou rcommunity for the first time and we found it’s very important for the community and it would be important if the implementation continues with a larger scale to reach many women. However, before implementing further awareness creation and health education should be given for the community through community mobilization.” (DC02)

“It was important because there were many women who had the chance of screening at their home without any payment. These women could have missed this chance as they couldn’t afford to pay for transportation and service fee and need to travel all such a long distanc to reach hospitals.” (DC03)

“In my opinion, it was good for the women to know whether they are free from this disease, but I don‘t think it’s a good option to take the sample at home. It would be better if women could come to a health facility and give the sample under the direct guidance of health professional while service providers could help these women to provide the sample based on the instructions given to them.” (DC04)

**Question 2: Could you share me how you felt to explain the self-sampling procedure for the participants?**

“For the first few days, we felt shy to explain for the woman as it was new. When the women were asking us what the instrument is, we felt shy to explain in a good way for the women. However, after a few days, we adapted to it and it was no more difficult to explain for the other women.” (DC01)

“First, when we went women’s home to explain how to use the instrument, women were asking us, what the instrument is and they feel uncomfortable to even hear about it and they thought to could hurt them while using. However, we approached them gently and told them as it couldn’t hurt them and explained the advantages of using the self-sampling instrument.” (DC03)

**Question 3: Why do you think some eligible women did not participate in the screening program?**

“There are misconceptions in the community since some of the community members considered the self-sampling instrument has male sperm and could impregnate women. This perception was not uncommenon especially in the first week of the study period but we could able to convince some of the women with the collaboration of community leaders and religious leaders. However, some women still were not willing to participate particularly wives of religious leaders. In addition, the Orthodox Christian followers who received the consubstantiation were not willing to participate and they said they don’t want to insert any foreign instrument into their vagina as it‘s

only created for their husband’s genitalia. They took the self-sampling instrument as it will defile them.”  (DC02)

**Question 3:  How do you describe the acceptance of home-based genital self-sampling?**

“It’t acceptable in the community and this is why we have collected sample from many of our mothers, how come we even collect 10 if they refused to use the instrument.”  (DC03)

**Probe: Do you think the community has accepted to use the instrument?**

“Not all of them, there are a lot of women who didn‘t take part in the study due to lack of knowledge. We have tried to convince those who didn‘t willing to participate by visiting their house to give them health education and we have convinced some of them, but there were women who totally didn‘t want to participate even after we visited their house 3 times and give them information.”  (DC03)

“The acceptance was good and most of them understood the purpose of the home-based self-sampling after we explained for them. However, misconceptions in the community concerning the self-sampling instrument has affected the acceptance among some women. Due to this reason there were women who afraid to sample themselves. Almost all the community support the idea, but even after we have visited their home for 3 times of health education some of the women were not willing to sample themselves due to the misconception in the community.” (DC05)

**Probe: How did the religious leaders perceive home-based genital self-sampling in this community?**

“We have Orthodox Christian followers and Muslims here in our community. The religious leaders support the use of genital self-sampling because they witnessed when women were dying because of lack of treatment in the community and they support the screening at home.” (DC02)

“Since no one knows whether it’s allowed or not by their religion that is why most of the women have accepted it. However, the women were checking one another who took the self-sampling or not, especially their neighbors.” (DC04)

**Question 4: Could you explain the participant’s feeling concerning home-based self-sampling?**

“Some women even provided the self-sample by requesting themselves, but some of them were not willing to provide it even after intensive health education. Because they didn’t see the instrument at a health facility and previously they have never participated in self-sampling at home. So they afraid the consequences of using this new device to sample themselves. Sometimes they explain their feeling saying it could hurt their genitalia and could cause bleeding, this is why some of them took the sampling instrument, but have never used it when they give it back.” (DC06)

**Probe: How do you think these misconceptions could be addressed?**

“During the previous sample collection, enough health education was not. The community didn’t get enough understanding and this is one of the reasons why women feel shy to use the instrument.” (DC06)

**Question 5: Have you faced challenges to explain how to use Evalyn brush® for the participants?**

“After they are willing to participate there was no problem by my side to explain the procedure to them. However, while some of the women easily understand the procedure, it‘s difficult for some of them. For those who couldn’t understand it easily, we explained it repeatedly. I have seen some women who were worried so much when we were showing them how to use the self-sampling instrument.” (DC03)

**Probe: Why do you think those women afraid to use the self-sampling instrument ?**

“I think they didn’t have any experience with the self-sampling instrument, so to prevent this the women should be familiar with the instrument during health education.” (DC03)

**Probe: Could you explain how you overcame when women did not understand the procedures?**

“Sometimes we went to their bedroom with them to show them how they should sample themselves. Also, if they don‘t felt confident in sampling themselves, we explained the procedure repeatedly for them.” (DC03)

**Question 6: Did you feel comfortable to visit women’s home to collect a sample?**

“I have faced challenges while collecting sample from some women since their husbands come during sample collection. For instance, while a woman was collecting a sample in her bedroom, I was waiting for her in the living room; suddenly her husband came into the home and asked what she was doing. I have explained as she is sampling herself for cervical cancer screening. Then he ordered her to come out from the bedroom and then she came out carrying the self-sampling instrument and he was so angry and said, ‘that means you have another husband without my knowledge[...]’, then he told us to leave his home immediately and we left the house.” (DC01)

**Probe: Did you ask the woman what he did to her after you left the home?**

“Yes we did, he told her not to participate in anything without consulting him. So, in my opinion, husbands should be educated everything about the screening and should sign the consent form with their wives.” (DC01)

“I my opinion, both husbands and wives should take health education and this she should be done until they understand it. The community should be educated as there is cervical cancer self-sampling and health educators should show them during health education.” (DC05)

**Question 7: Could you describe how other family members react when you visit the women’s house to collect a sample?**

“In the first place some of the family members didn’t have a good attitude for this screening, they were saying, for our wives they brought other husband and were laughing. But later on, they understood. I remember a husband chased us with his dog saying my wife should never take this screening due to lack of knowledge. So community needs health education .” (DC02)

“In my opinion, husbands should be involved in health education and informed as their wife is taking part in the screening. First of all, husbands should believe as the screening has benefit for their partner unless it’s difficult for women to participate. For instance, a man has chased us from his home while we were providing health education for his wife, saying, ‘what kind of screening is that you are giving to my wife without consulting me ?’ So, I say health education should be given to the husbands of every woman who participates in the home-based genital self-sampling.” (DC04)

**Probe: Do you think it is important if you educate both a husband and wife together ?**

“During the training, we were told as we should talk to the mother alone when we are explaining the procedure to her and that may decrease her fear. Once I went one home and husband and wife were sitting together. I gave them health education and told the husband to leave a room to show the sampling instrument to his wife, but he insisted to know what I was showing her. So in my opinion, everything concerning self-sampling should be explained for the couples.” (DC04)

**Question 8: Do you think the women had family support to participate in the self-sampling study?**

“I think some of the women had support from the family, for instance, there was a husband we told us to go his home and sample his wife when he saw us on the road side. For me, these husbands are supporting their wives.” (DC06)

**Probe: Do you think husbands should have to know the participation of their wives?**

“Yes, husbands must know and participate in health education. Spouses in the rural area are not openly discussed with each other and I think they afraid to talk together about private parts and women fear her husband [...]. Some of the women whose husband didn’t participate in health education of self-sampling were insulted by their husbands after they told their husbands they participated in the study without their husband’s knowledge.” (DC06)

**Theme 2: Barriers to the provision of quality sample for HPV testing**

**Question 1. What do you think were the challenges for the women to provide a sufficient sample for HPV testing?**

“Maybe we as a sample collectors couldn’t go further to make the women understand the ginital self-sampling procedure and may give the instrument for a woman before she understands it. For instance, after we showed them how to use the instrument and they are clear on how to use the instrument, they they forgot every step when you go to private place to sample themselves and we showed them again all the procedures. Sometimes when women face challenges to understand the instructions, we went to their bedroom with them and showed them how they should stand, and rotate the instrument.” (DC04)

“In my opinion, there is no problem on the side of sample collectors because we went to a field after we were able to show all the procedures and getting enough understanding. However, in my

opinion, the problem is from the women because after we explained all the procedures they went to their bedrooms to sample themselves. After that we collected what they gave us, we were told not to check the qulaity of collected sample. So, we were not sure whether the woman has used the instrument or not. So, the problem could be from women's side since they mau not understand the procedure well or didn't want to sample themselves after collecting the instrument." (DC03)

"The time given for the sample collection was limited and we didn't get enough time to provide detailed health education. Even we have seen the instrument for the first time and you can imagine how it could be difficult to use it for the rural women to use it without repetitive health education.

In addition, some of the women didn't understand the procedure easily and we spent a lot of time showing them how to use it. The main challenge was some of the women considered the instrument could hurt their genitalia." (DC01)

**Question 2: How did you identify whether the women have used the instrument (Evalyn brush®)?**

"For the first few days of sample collection period, the women were giving us the self-sampling instrument before they close it and we were able to check as they have used it, and when they didn't use it propely, we told them to go back and sample themselves. However, we were told by the trainers to collect a closed instrument from the women. So there was no method of checking whether a woman has given us sufficient sample or not, even it isn't possible to identify whether she has used the instrument or not." (DC05)

"If you may listen to her attentively, you could hear the sound kua kua [.... ] five times when she uses the instrument. That is the way I was checking as the women were using the instrument." (DC01)

**Theme 3: Suggestions of the participants to guarantee the success of home-based HPV self-sampling.**

**Question: Do you have any suggestion on how to improve the quality of a home-based genital self-sample for HPV testing?**

"As I said earlier, health education should be improved and given to all the community since our rural population don't have any information about this self-sampling. I believe this will improve

the acceptability of cervical cancer screening in this community. In addition, women with abnormal findings were not linked to to further follow-up and some of those who provided sample for second time didn’t receive their result yet. I think these problems should be solved to increase the acceptibility of the screening in the communiy.” (DC02)
