# Supplementary material for: Community cervical cancer screening: Barriers to successful home-based HPV self-sampling in Dabat district, North Gondar, Ethiopia. A qualitative study
Source: PLoS One. 2020 Dec 11;15(12):e0243036. doi: 10.1371/journal.pone.0243036 (PMC7732077; doi:10.1371/journal.pone.0243036)
Supplement: Attachment — Submitted filename: Review Community cervical cancer screening PLOS ONE1 (2).docx [file pone.0243036.s002.docx]

**Review PLOS ONE - PONE-D-20-06352**

**Community cervical cancer screening: Barriers to successful home-based HPV self-sampling in Dabat district, North Gondar, Ethiopia. A qualitative study.**

This is a collaborative study between research groups in Ethiopia and Germany to assess the barriers to carrying out self-sampling of the vaginal canal at home to identify HPV as a cervical cancer screening strategy.

The main findings can be attributed ot sociocultural factors and indicate that the main barriers are the husband’s attitude, the social environment, lack of knowledge of cervical cancer and its etiology; and to absence of recommendation and acceptance of this practice by health workers as a whole and by localo community leaders.

The manuscript as such is a pilot study that uses a methodological strategy of in-depth interviews with 22 women and the development of 4 focus groups of 6-7 women each. It is widely described although the information is not very original and recommendations are few.

The information obtained is, again, not original. The results obtained are similar to those reported in the 1980s on barriers to the use of cervical cytology in poor countries, as well as on the acceptability of HPV testing and vaginal canal autotomy since the late 1990s.

Concern for the correct use of vaginal auto-collection instruments and the feeling of shame with male partners and health professionals is a fact of life in most cultures. The manuscript is generally very descriptive, with very limited recommendations for the context of a geographic area of very low cervical cancer screening coverage. Although the manuscript was written to describe barriers to utilization, it would have been advisable to list possible interventions to improve acceptability and how to address low healthcare coverage in population-based screening programs.
